# Multispectral imaging of nailfold capillaries using light-emitting diode illumination

**DOI:** 10.1117/1.JBO.27.12.126002

**Published:** 2022-12-12

**Authors:** Michaela Taylor-Williams, Stephen Mead, Travis W. Sawyer, Lina Hacker, Calum Williams, Michael Berks, Andrea Murray, Sarah E. Bohndiek

**Affiliations:** a University of Cambridge, Department of Physics, Cavendish Laboratory, Cambridge, United Kingdom; b University of Cambridge, Cancer Research UK Cambridge Institute, Cambridge, United Kingdom; c University of Arizona, Wyant College of Optical Sciences, Tucson, Arizona, United States; d University of Manchester, Division of Cancer Sciences, Quantitative Biomedical Imaging Laboratory, Manchester, United Kingdom; e University of Manchester, NIHR Manchester Biomedical Research Centre, Manchester, United Kingdom

**Keywords:** multispectral imaging, nailfold capillaroscopy, light-emitting diode (LED), oximetry, hemoglobin, phantom, illumination modeling, spectral modeling

## Abstract

**Significance:**

The capillaries are the smallest blood vessels in the body, typically imaged using video capillaroscopy to aid diagnosis of connective tissue diseases, such as systemic sclerosis. Video capillaroscopy allows visualization of morphological changes in the nailfold capillaries but does not provide any physiological information about the blood contained within the capillary network. Extracting parameters such as hemoglobin oxygenation could increase sensitivity for diagnosis and measurement of microvascular disease progression.

**Aim:**

To design, construct, and test a low-cost multispectral imaging (MSI) system using light-emitting diode (LED) illumination to assess relative hemoglobin oxygenation in the nailfold capillaries.

**Approach:**

An LED ring light was first designed and modeled. The ring light was fabricated using four commercially available LED colors and a custom-designed printed circuit board. The experimental system was characterized and results compared with the illumination model. A blood phantom with variable oxygenation was used to determine the feasibility of using the illumination-based MSI system for oximetry. Nailfold capillaries were then imaged in a healthy subject.

**Results:**

The illumination modeling results were in close agreement with the constructed system. Imaging of the blood phantom demonstrated sensitivity to changing hemoglobin oxygenation, which was in line with the spectral modeling of reflection. The morphological properties of the volunteer capillaries were comparable to those measured in current gold standard systems.

**Conclusions:**

LED-based illumination could be used as a low-cost approach to enable MSI of the nailfold capillaries to provide insight into the oxygenation of the blood contained within the capillary network.

## 1 Introduction

Nailfold capillaroscopy is a noninvasive imaging modality used to evaluate nailfold capillary morphology and pathological changes of the finger.^1^ Capillaroscopy aids the clinical diagnosis and monitoring of rheumatological diseases, such as systemic sclerosis (SSc), also known as scleroderma.^2^ In particular, capillaroscopy forms part of the classification criteria used to diagnosis SSc.^3^ Video capillaroscopy, which is the gold standard in nailfold capillaroscopy, combines an optical microscope and digital camera to magnify the capillaries so that clinicians can accurately measure their morphology to assess abnormality (e.g., enlarged capillaries and decreased density),^4^^–^^6^ yet it fails to reveal the functional changes associated with capillary deformations.^1^^,^^7^ Microvascular deformation observed at the nailfold in SSc is hypothesized to arise due to hypoxia, but this has proven difficult to measure. Low oxygen is theorized to be a key element of the disease process; superficial skin changes are thought to mirror underlying changes occurring in internal organs.^8^ Understanding how blood oxygen saturation is related to microvascular deformation, and the role it plays in the development and progression of connective tissue disorders could improve disease diagnosis, monitoring, and treatment.^9^^,^^10^

Hemoglobin, the protein that binds and transports oxygen around the body, is the primary optical absorber in blood.^11^^,^^12^ Hemoglobin exhibits distinct spectral absorption characteristics depending on its oxygenation state,^13^ hence measurement of the relative oxygenated (${\mathrm{HbO}}_{2}$) and deoxygenated (Hb) hemoglobin concentrations can be used to calculate blood oxygen saturation (${\mathrm{sO}}_{2}=[{\mathrm{HbO}}_{2}]/[\mathrm{Hb}+{\mathrm{HbO}}_{2}]$). Oxygen saturation can be optically measured in the finger using well-established pulse oximetry;^14^^–^^16^ however, oximetry relies on pulsatile blood flow and does not allow for visualization of the oxygenation dynamics in combination with morphological deformations or perfusion measurements. To gain a greater understanding of the interplay between microvascular deformation and oxygen saturation, a spatial mapping of the oxygenation information is necessary, which can be achieved using spectral imaging methods.^12^^,^^17^^,^^18^

Spectral imaging refers to the measurement of optical absorption as a function of wavelength at every pixel within the image.^19^^,^^20^ The relative concentration of ${\mathrm{HbO}}_{2}$ and Hb can be extracted non-invasively from recorded spectra based on their distinct absorption characteristics using computational methods, such as spectral unmixing.^21^ The resulting abundance maps could be overlaid onto the standard video capillaroscopy images combining morphological and physiological information.^20^ Spectral imaging hardware can be broadly classified into spatial-scanning, spectral-scanning, and snapshot methods.^12^^,^^22^ Spatial-scanning methods are impractical in capillaroscopy, given the requirement for video-rate measurement and the challenging topology of the finger.

Snapshot methods typically require a trade-off between spatial and spectral resolution,^23^ whereby spatial resolution often degrades with an increasing number of spectral bands,^24^ which can be challenging in capillaroscopy given the need to make detailed morphological assessments of the capillary structures at high resolution. Customized multispectral filter arrays may overcome this challenge by reducing the number of required spectral bands, but these have yet to be demonstrated for accurate quantification of blood oxygenation.^24^^,^^25^ Spectral-scanning methods can vary in complexity can cost, employing either tunable illumination or imaging filters, however, such systems can be large and expensive.^22^ Alternatives, such as Fourier transform spectroscopy and optical coherence tomography (OCT), are also capable of measuring optical absorption, ^12^^,^^26^ however, increased complexity hampers rollout and integration in a clinical setting.^27^

We hypothesized that imaging with a limited number of spectral bands could alleviate the challenge of achieving video-rate deployment while adding a sufficient spectral dimension to the data to obtain oxygenation-related information. Such multispectral imaging (MSI)^12^^,^^19^^,^^28^^,^^29^ can retain high accuracy for spectral unmixing when the imaging scene only contains a few spectrally unique constituents. MSI is also advantageous when real-time capture is essential, compactness is required, or low system cost is paramount.^19^ Moreover, in a biological setting where the reflectance spectra are smoothly varying, near-continuous spectral sampling often provides minimal additional information compared with a carefully selected set of discrete (multispectral) bands.^19^^,^^24^^,^^25^^,^^30^^,^^31^

To this end, we designed and built a cost-effective light-emitting diode (LED)-based multispectral illumination system with readily available commercial components to test the potential of spectral-scanning MSI in capillaroscopy. We first compared the differential absorption spectra of Hb and ${\mathrm{HbO}}_{2}$ with commercially available LED colors. We next designed, modeled, and fabricated a custom LED-based ring illuminator containing a range of LED colors, including three types of narrowband LEDs (center wavelengths: 505, 572, and 592 nm) and one type of white light LED. The optical properties of the LED ring were then characterized and experiments performed in a controlled blood phantom system before imaging the finger of a healthy volunteer. Our findings indicate that an LED-based approach could potentially add functional information to capillaroscopy systems in the future. With further development, MSI capillaroscopy could be tested for the improvement of SSc diagnosis and treatment.

## 2 Methods

### 2.1 Reflectance Modeling and LED Selection

The expected difference in reflectance of blood with 80% and 100% ${\mathrm{sO}}_{2}$^32^ was evaluated and used to select the LED center wavelengths and bandwidths for illumination. 100% ${\mathrm{sO}}_{2}$ was taken as the upper limit because arterial blood typically has between 96% and 100% ${\mathrm{sO}}_{2}$.^11^^,^^16^ 80% was taken as the lower limit because the venous oxygen saturation of the finger and hand has been reported to range from 80% to 86.2% in previous studies of blood oxygenation in the hand.^32^

**Fig. 1:**
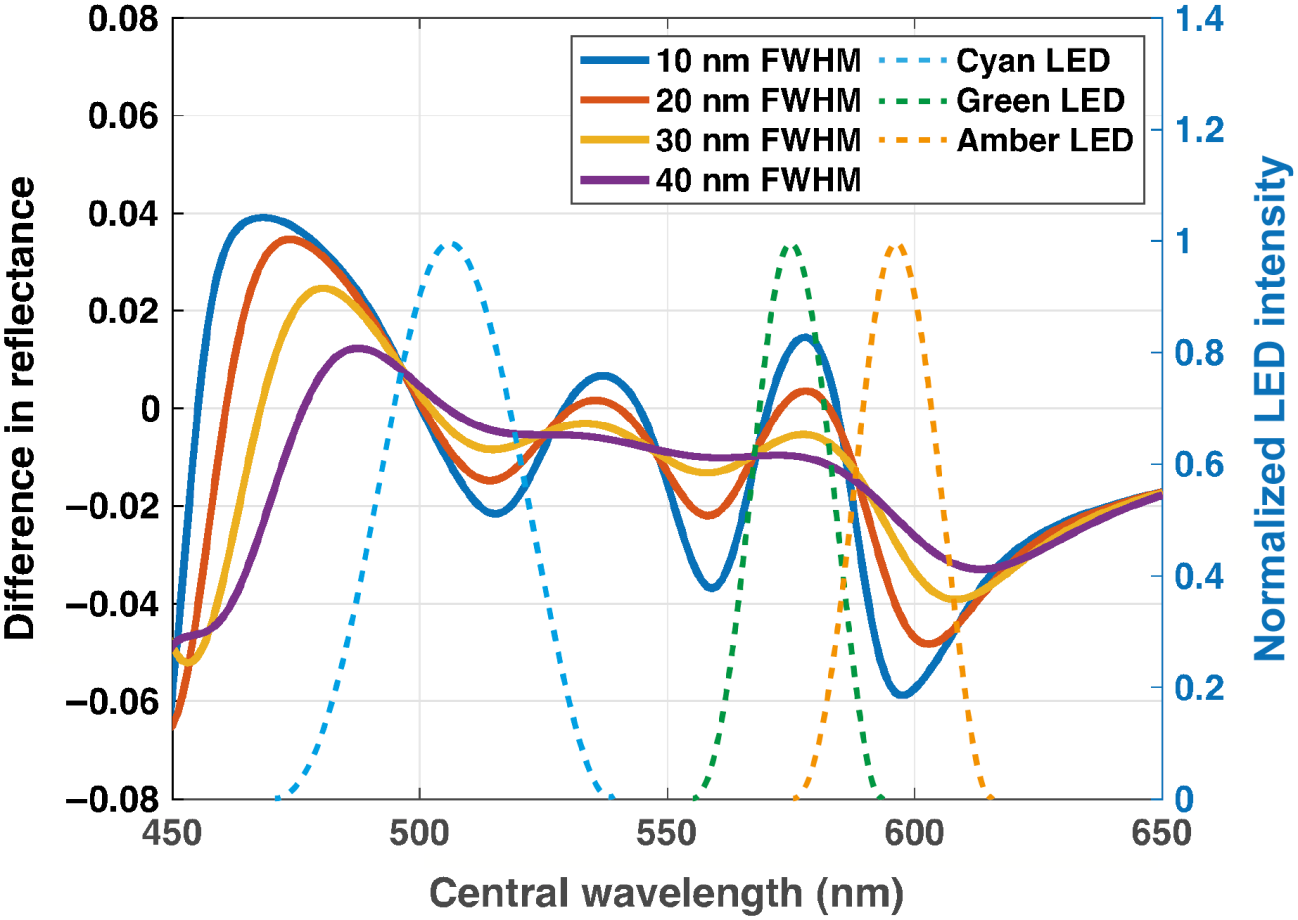
The calculated difference in reflectance between 100% and 80% oxygenated hemoglobin was used to enable LED selection. Data shown for four modeled bandwidths: 10 nm (blue), 20 nm (orange), 30 nm (yellow), 40 nm (purple), and all solid lines. The three selected LED intensity profiles (cyan, green, and amber; Table 1, all dotted lines) are overlaid for reference and correspond to the right axis.

LED spectral irradiance distributions were represented as 200 Gaussian functions with center wavelengths between 450 and 650 nm in 1-nm steps; eight different bandwidths were then tested (measured as the full width half maximum, FWHM) between 10 and 40 nm in 5-nm steps (only four different bandwidths are shown in Fig. 1 for simplicity). Each Gaussian from 450 to 650 nm with 1-nm steps was normalized so that the area under the curve was equal to one and multiplied first by the absorption spectrum^33^ of either 80% or 100% ${\mathrm{sO}}_{2}$ and then by the quantum efficiency of a monochrome silicon-based complementary metal-oxide-semiconductor image sensor (data taken from Basler a2A3840-45umPRO, available on request from manufacturer).

The predicted sensor signal was then integrated over all wavelengths to give the total absorbance (A) expected for a given central wavelength and bandwidth of illumination. The A was then converted to the total reflectance (R) as^34^

$$R(\lambda )=10^{-A(\lambda )},$$ (1)

where λ is the wavelength.

The calculated difference in reflectance for the conditions of 80% and 100% ${\mathrm{sO}}_{2}$ (Fig. 1) was used to guide the selection of LEDs (Table 1) based on selection criteria including: center wavelength; bandwidth; illumination power; viewing angle; electrical properties (voltage and current requirements); availability; and cost. To avoid unnecessary exposure to ultraviolet and blue light, which can induce photodamage,^35^^,^^36^ center wavelengths considered were restricted to those >450  nm. Many available LEDs did not have a bandwidth <20-nm FWHM and few had a central wavelength in the 520 to 560 nm range. Further, few LEDs in this range have sufficient illumination output to provide adequate illumination power in the sample plane. Taken together, these considerations led to the selection of three narrowband LEDs and one broadband white LED (Table 1), aiming respectively to interrogate one isosbestic point and two regions where either oxygenated or deoxygenated hemoglobin dominated the absorption, and to provide a reference color image.

**Table 1:** Spectral properties of the LEDs used in the system and the difference in the calculated reflectance.

Color	Product, vendor	Central wavelength (nm)	FWHM (nm)	Difference in reflectance (a.u.)	Aim
Cyan	HLMP-CE34-Y1CDD, Avago	505	30	−0.00327	Isosbestic
Green	TLCYG5100, Vishay	572	15	0.00239	${\mathrm{HbO}}_{2}>\mathrm{Hb}$
Amber	TLCY510, Vishay	590	17	−0.02310	$\mathrm{Hb}>{\mathrm{HbO}}_{2}$
White	Warm white, Multicomp	NA	NA	NA	Gold standard for RGB imaging

### 2.2 LED Ring-Light Design and Modeling

**Fig. 2:**
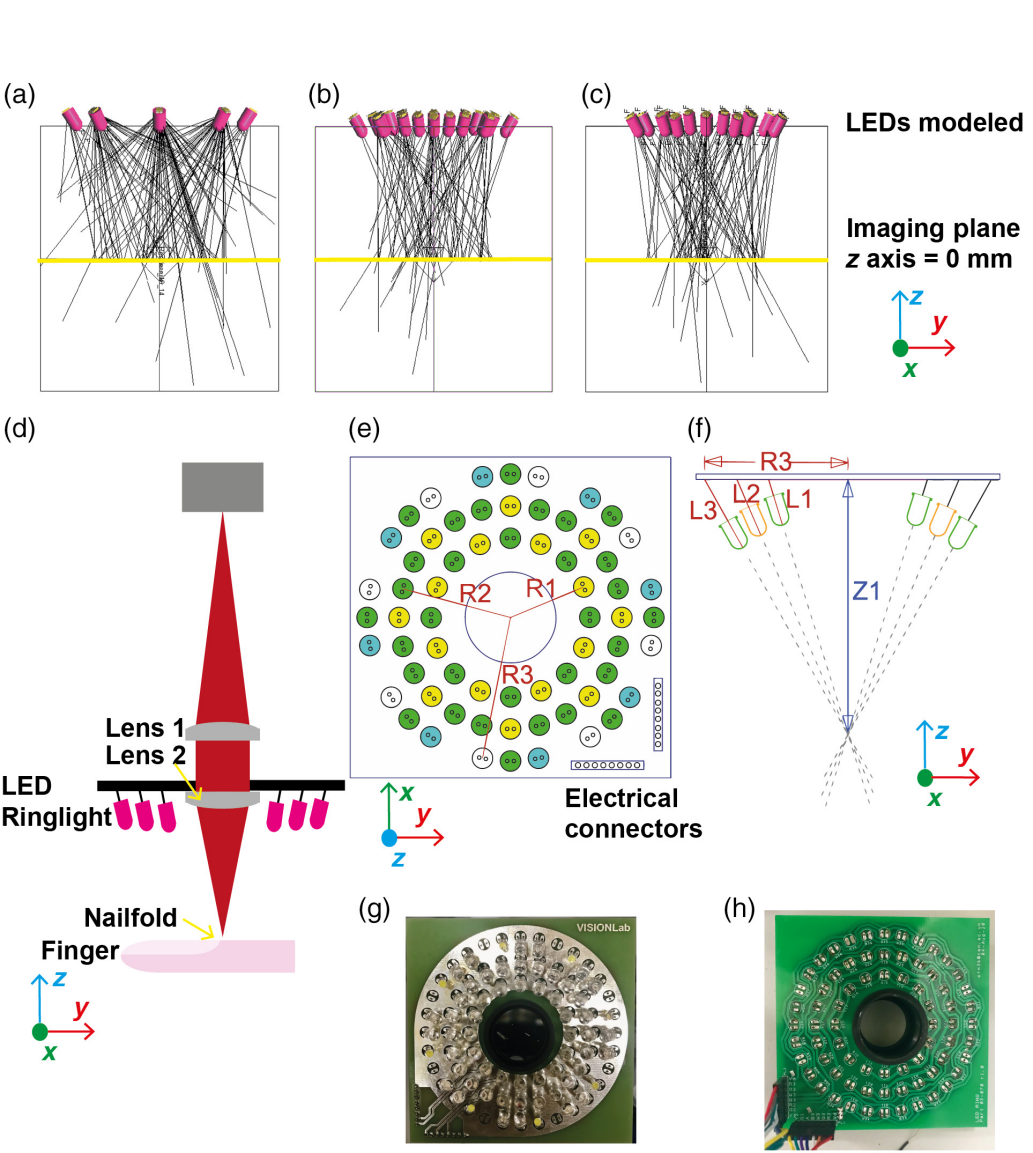
Multispectral illumination system based on a custom LED ring light. The LED arrangements for cyan (a), green (b), and amber (c) light that were modeled in LightTools, normal to the imaging axis. The yellow line denotes the imaging plane at the z axis of 0 mm, and the black lines represent a subset of rays from the distribution used to simulate the illumination. (d) The nailfold is illuminated by the LED ring light, and the image is magnified by achromatic doublet lenses (lens 1: f=120  mm, lens 2: f=60  mm); the camera then detects the image with a monochrome sensor. The axis used in modeling and characterizing the system is demonstrated. (e), (f) The LED colors are arranged symmetrically on a PCB (100  mm×100  mm) with the LEDs manually angled towards the optical axis at the object plane that was 67.2 mm away (Z1). The radii R1, R2, and R3 were 23.50, 33.02, and 42.55 mm, respectively, with legs lengths of L1, L2, and L3 that were 13.1, 13.7, and 14.6, respectively. (g), (h) Photos of the constructed PCB with LEDs and connectors.

The system was first modeled using LightTools 9.1.0 (Synopsys, United States) to determine the expected spatial illumination properties of the system [Figs. 2(a)–2(c)]. Different geometries of the illumination and placement of the LEDs were considered, such as a ring light on a flat surface, ring light on a curved surface, or illumination dome. The illumination dome was discounted as it would be difficult to accurately position a finger in such a system, and the curved surface ring light presented fabrication difficulties. A flat surface, such as a circular printed circuit board (PCB) with LEDs, was simpler to fabricate and could easily be integrated into clinical capillaroscopy systems. The LED properties (viewing half-angle, center wavelength, optical power, physical dimensions, and orientation) were used as input parameters for the modeling. The illumination propagation from the LED array to the focal plane [Fig. 2(d)] was modeled to investigate the spatial uniformity of the irradiance distribution. The number of LEDs for each color was selected to balance the image illumination given their illumination power, assuming a constant frame rate of acquisition for each color. The fixed geometrical properties of the ring light model included: a large diameter of 30 mm and up to 72 LEDs to achieve a high illumination level on the sample; LED arrays raised from the PCB and tilted towards the center of the focal plane; and 60 mm working distance to permit easy movement of the finger under the optical system [Figs. 2(e) and 2(f)].

The LED ring light was then deployed experimentally in a nailfold capillaroscopy system [Fig. 2(d)]. The optical magnification of the lens assembly was 2.5× with a 60-mm working distance. Two achromatic doublets with focal lengths of 150 and 60 mm were used (ThorLabs AC254-060-A-ML and AC254-150-A-ML). A 12-bit monochrome camera (Basler ace2 a2a3840-45umpro) with a varying frame rate was used to capture images of the nailfold. The LEDs were arranged in three circles of different radii (R1, R2, and R3) on the PCB (Fig. S1 in the Supplementary Material) to create a custom ring light, radially centered to a 26.8-mm diameter hole specified to accommodate 1-inch diameter optical components [Fig. 2(e)]. LEDs were arranged symmetrically and pointed toward the center of the imaging plane [Fig. 2(f)]. The imaging plane was located 67.2 mm from the PCB, indicated by Z1 [Fig. 2(f)]. The radii of the LED arrangement R1, R2, and R3 were 23.50, 33.02, and 42.55 mm, respectively. The LEDs were offset from the PCB such that the lengths of their legs L1, L2, and L3 were 13.1, 13.7, and 14.6 mm, respectively. The temporal variation in the LED irradiance for each color was measured (Fig. S2 in the Supplementary Material). The spectral properties of the LEDs were verified independently (Figs. S3, S4, and Table S1 in the Supplementary Material).

### 2.3 Illumination Spatial Uniformity Characterization

The illumination distribution of the LED ring light was measured by capturing the propagated light at two orthogonal planes: (1) tangential to the optical axis (z axis) centered about the object plane (x–z, y=0  mm) and (2) the object plane (x–y, z=0  mm). A 1-inch (diagonal) sensor (FLIR Grasshopper Camera: *GS3-U3-41C6M-C*) was used for this experiment; the casing was removed to avoid vignetting and the linearity established according to the methods described in Sec. 2.4 to ensure that imaging data from across the object plane could be stitched together. Mechanical translation stages (Thorlabs: MTS50/M-Z8 and MTS25/M-Z8) were used to scan both the x and y axis in steps of 10 mm. For the x–z plane measurements, seven incremental steps along the z axis between 10 and 44 mm were used. Measurements were repeated for each LED color. Single point power measurements along the z axis at 2-mm increments (centered in the x axis) were obtained using a calibrated photodiode power meter (Thorlabs: S121C), which allowed the results to be normalized to their peak power to compare with the modeled power.

### 2.4 Sensor Characterization

The sensitivity and linearity of the monochrome cameras used in this study (Secs. 2.1 and 2.3) were measured in accordance with EMVA Standard 1288^37^ (Fig. S3 in the Supplementary Material) to confirm that a change in measured reflectance could indeed be treated as a linear change in sample absorption. For each exposure time $t_{\exp}$, the mean grey values, such as $\mu_{y}$, and $\mu_{y.\text{dark}}$, and the temporal variance of the grey values, ${\sigma_{y}}^{2}$ and ${\sigma_{y.\text{dark}}}^{2}$, were calculated in all images. To avoid the effects of vignetting, the values were computed over an area of 1024×768  pixels in the center of the image only. The photon transfer curve of the variance against the mean values was used to obtain the system gain, K, from a linear regression line for values between 0% and 70% of saturation. The slope of the sensitivity curve allowed an estimation of photon flux $\mu_{p}$ from^37^

$$\mu_{p}=\frac{aEt_{\exp}}{h(\frac{c}{\lambda })},$$ (2)

where a is the pixel area, h is Planck’s constant, and c is the speed of light in a vacuum, and E is irradiance taken as the value of quantum efficiency from the manufacturer data sheet. The signal-to-noise ratio (SNR) for the sensor was then used to determine the noise equivalent irradiance (SNR=1) to assess the suitability of the sensor for multispectral reflectance measurements. The linearity of both sensors was confirmed over the operating range of our experiment (Figs. S5 and S6 in the Supplementary Material).

### 2.5 Blood Phantom Experiment

A blood flow phantom was used to test the ability of the LED-based MSI system to detect changes in oxygenation. The blood flow apparatus comprised a clear polydimethylsiloxane (PDMS) phantom on top of a titanium dioxide (${\mathrm{TiO}}_{2}$) PDMS base, included to promote reflectance. The channel imaged was rectangular with a width of 1 mm and depth of 32.3  μm. Horse blood in ethylenediaminetetraacetic acid (EDTA) (EDTA; TCS Biosciences, HB073) was initially oxygenated using hydrogen peroxide (7722-84-1, Sigma-Aldrich) by adding 0.2% v/v, and separately deoxygenated using sodium hydrosulfite (A9539-500G, Sigma-Aldrich), which deoxygenated the blood from 99% to 0% ${\mathrm{sO}}_{2}$. The sodium hydrosulfite was dissolved in phosphate-buffered saline (PBS) at ∼0.06 w/v% before mixing it with the blood at 4% v/v. Experiments were performed at room temperature. Oxygenation probes (OxyLite Pro, Oxford Optronix) were used to measure the oxygen pressure (${\mathrm{pO}}_{2}$) of the blood. To convert measured ${\mathrm{pO}}_{2}$ values into ${\mathrm{sO}}_{2}$ values, the Severinghaus equation ^38^ was used. Initially, a syringe with 99% ${\mathrm{sO}}_{2}$ blood was pumped through the microchannel using a syringe pump (MKCB2159V, Harvard) at a rate of 1.8  mL/min. This corresponds to an average velocity of 1  mm/s, within the range of blood cell velocity measured in previous work: 0.5 to 6.8  mm/s.^39^ Next, a syringe with 0% ${\mathrm{sO}}_{2}$ blood was used, and finally, the system was flushed with PBS to take reference measurements. The LEDs were turned on for at least 10 min to ensure temporal stability prior to image acquisition (Fig. S2 in the Supplementary Material). Forty images at each illumination were taken over a period of 200 seconds with camera exposure times of 2.001, 10.002, and 2.496 ms for the cyan, green, and amber illuminations, respectively. These correspond to frame rates of 500, 100, and 400 fps, respectively, when operated in a continuous manner. These were selected to optimize the contrast between the blood and surrounding phantom, so neither region was oversaturated.

The 40 images taken under each illumination were collated into a single image by finding the median value of each of the pixels within the set to avoid artifacts arising from small bubbles that can flow through in the microfluidic channel in different frames. The final image was then divided by the reference image taken of PBS in the phantom to normalize the reflectance. The reflectance values were averaged over six regions of interest in the 0% and 99% ${\mathrm{sO}}_{2}$ phantom images. A two-tailed Student’s t-test was performed to evaluate the significance of the results.

### 2.6 Nailfold Imaging

The finger nailfold was coated in castor oil for index matching and placed underneath the ring light system. The finger was kept stationary using a three-dimensional (3D)-printed finger mold. The mold was printed with polylactic acid using an Ultimaker S3.

### 2.7 Image and Statistical Analysis

All analysis code was written in MATLAB 2020 unless otherwise stated. Sensor characterization measurements were analyzed using custom code based on the EMVA 1288 standard. Images measured to assess spatial illumination uniformity were stitched together in Fiji (ImageJ) using the macro plugin Grid/Collection,^40^ and subsequently, spline interpolated using custom code to create cross-sectional images for comparison to the modeling results. Agreement between measured and modeled results was tested by calculating the root mean squared error (RMSE) values. For blood phantom measurements, a region of interest (ROI) of 100×100  pixels within the image of the blood tubing was averaged to extract the spectral data. Statistical significance was tested using a two-tailed Student t-test.

Quantitative analysis of nailfold capillaries involved calculation of the average values of capillary signal, background, contrast, and SNR. After white field correction, the central regions of the individual capillaries were extracted where the loop edge was clearly defined, and calculations were made in this region. The capillary regions were analyzed by extracting the horizontal line profile to find the average signal, $\overset{¯}{s(y)}$, and background signal, $\overset{¯}{b(y)}$. The SNR was then calculated according to

$$\mathrm{SNR}(y)=\frac{\overset{¯}{s(y)}-\overset{¯}{b(y)}}{b_{\sigma }(y)},$$ (3)

where $b_{\sigma }(y)$ is the standard deviation of the background signal. These values were averaged throughout the chosen central capillary region to calculate the average signal, background, and SNR for each capillary. Capillaries (n=23) on each finger (n=3; digits 2, 3, and 4) were selected and their morphological differences in length and width were calculated. The relative oxygenation map was prepared using the cyan and amber LED images. Three select capillaries that were in focus and did not overlap with other capillaries were cropped from the amber and cyan illumination. The two capillary images were first aligned in Fiji (ImageJ), using the macro plugin Big Warp,^41^ and the resulting amber illuminated image was divided by the cyan illuminated image. The resulting images were then normalized so that the maximum relative oxygenation was 1.0. Different methods of calculating the relative oxygenation are explored in the Supplementary Material (Fig. S8 and Table S3 in the Supplementary Material).

## 3 Results

### 3.1 Irradiance Profiles are Consistent Between Modeling and Measurement

**Fig. 3:**
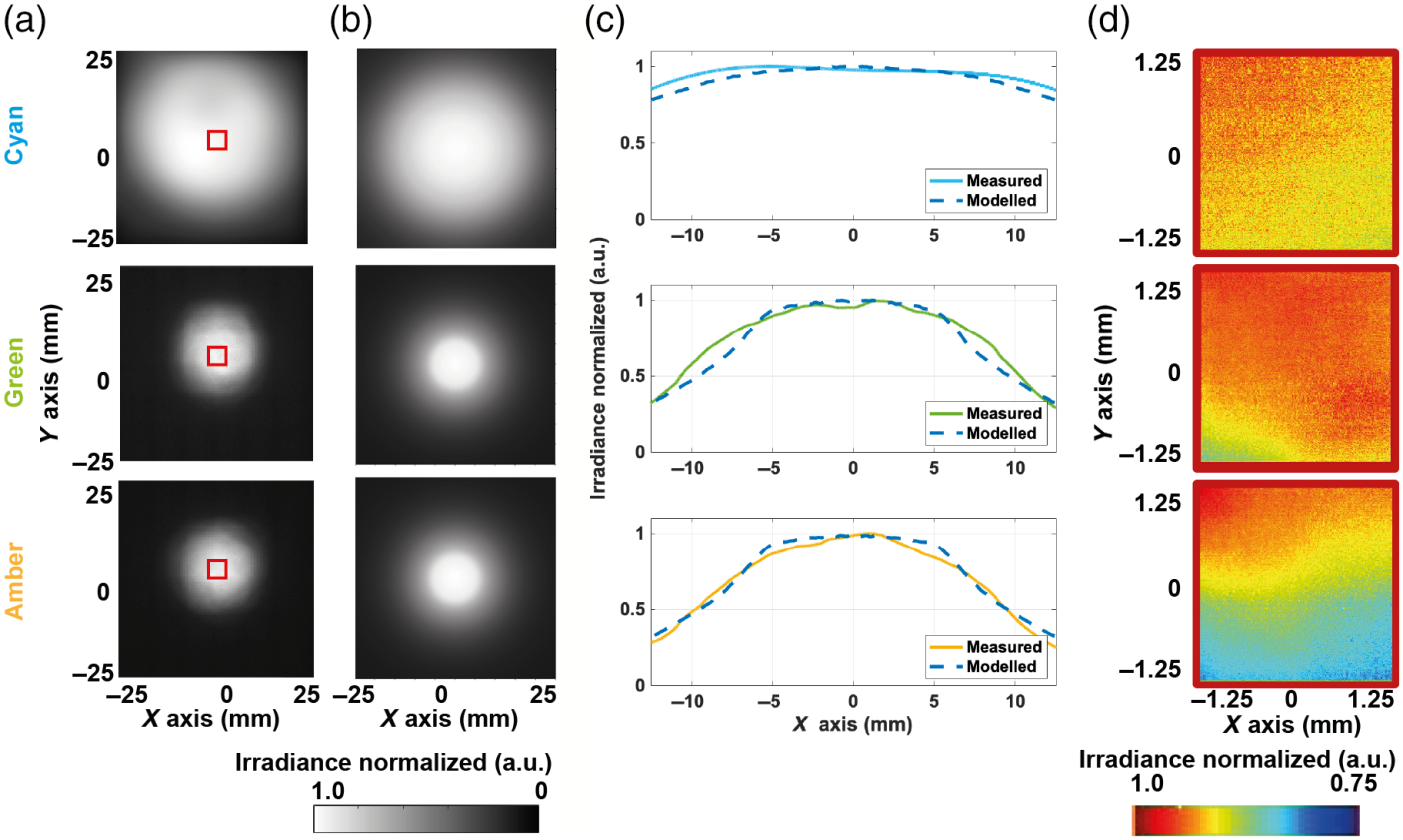
Comparison of the measured and modeled irradiance in the object plane. Comparison of uniformity at the object plane (z=0  mm) of the (a) measured and (b) modeled irradiance for cyan (top), green (middle), and amber (bottom) LED light. (c) Line profiles along the x axis of the measured and modeled system. (d) Examination of a realistic ROI of ∼2.5  mm×2.5  mm, as illustrated in panel (a) (red square).

Understanding illumination uniformity is vital in MSI to avoid artifacts in the collected spectra^42^ and enable quantitative spectral unmixing. We first examined the experimentally realized illumination in the object plane (x–y; z=0  mm, Fig. 3) across a 50  mm×50  mm area to encompass the entire LED irradiance. Qualitative comparison with that predicted by modeling shows a visually good agreement between the modeled and measured illumination [Figs. 3(a) and (b)], which is also seen in the quantitative comparison [Fig. 3(c)]. Due to the high magnification of capillaroscopy systems, the field of view of the system would typically be <2.5  mm×2.5  mm, so a subregion in the center of the object plane was further analyzed [Fig. 3(d)]. A uniformity >75% of the maximum irradiance was found for all colors, with the best performance achieved with the cyan LEDs, despite being relatively fewer in number. This is probably due to the higher viewing angle of the cyan LEDs, resulting in less drop-off of the irradiance across the object plane. Spatial nonuniformities observed suggest that care should be taken in white field correction of collected data prior to quantitative analysis.

**Fig. 4:**
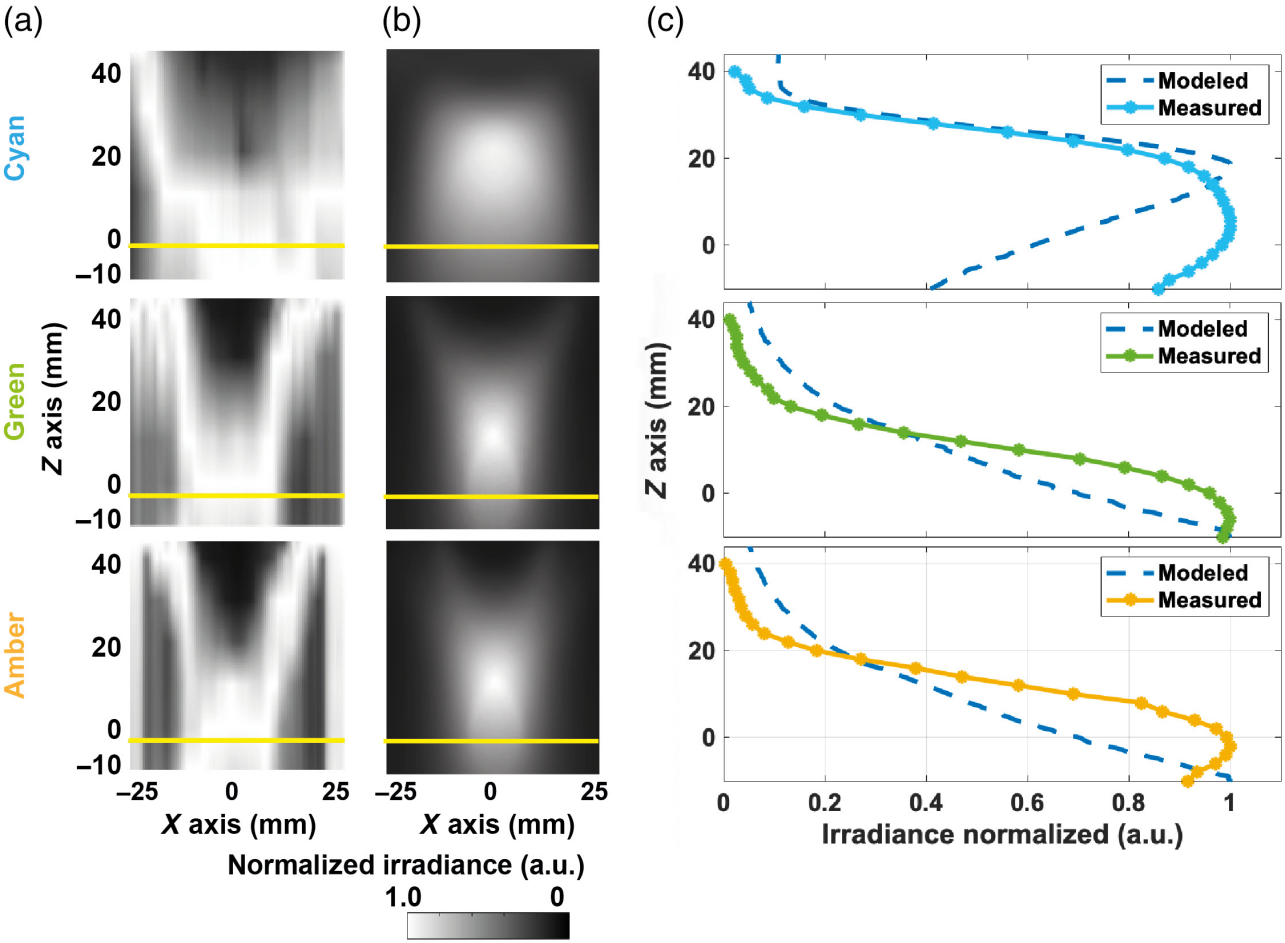
Comparison of the measured and modeled cross-sectional (x–z) irradiance of the LED ring light. The measured cross-section irradiance profiles (a) were compared with the initial model (b). The yellow line indicates the object plane (z=0  mm) for which data are presented in Fig. 3. (c) Quantification of the normalized optical power along the z axis is displayed for the model and measured values from the experimental system.

A similar exercise was then undertaken in the x−z plane (Fig. 4). The small asymmetries observed in the measured data [Fig. 4(a)] are likely due to misalignment of the LEDs in the experimental ring light compared with the model, because LEDs were angled by hand. Qualitative comparison with that predicted by modeling [Fig. 4(b)] showed a deviation in the position of the peak intensity, which was confirmed in the analysis of the normalized optical power delivery [Fig. 4(c)]. In particular, for the cyan LED, the model indicated the peak intensity would occur 18 mm past the imaging plane, which when measured, showed the peak intensity occurring at only 4 mm in front of the imaging plane. Subsequent remodeling performed by systematically changing the parameters for the LEDs suggested that the discrepancy between the experiment and the model might arise due to differences in alignment of the LEDs and the actual viewing angle of the LEDs compared with the datasheet values input to the model (Table S2 and Fig. S7 in the Supplementary Material). The modeled and measured systems were also quantitatively compared by comparing the x and z axis using RMSE as a metric (Table 2). Good agreement is demonstrated along the x axis for all colors, whereas the model also shows better agreement in the z axis for the green and amber illumination than the cyan; this is explored further in Table S2 and Fig. S7 in the Supplementary Material. These observations agree with the qualitative comparisons shown in Figs. 3 and 4.

**Table 2:** The modeled LED light systems were compared with the measured system in both the z and x axis using RMSE to illustrate the difference in models.

Original systems compared	Viewing angle (deg)	Z axis RMSE	X axis RMSE
Cyan	30	0.235	0.042
Green	15	0.141	0.065
Amber	15	0.177	0.041

### 3.2 LED-Based MSI of Blood Phantoms Shows Sensitivity to Oxygenation

**Fig. 5:**
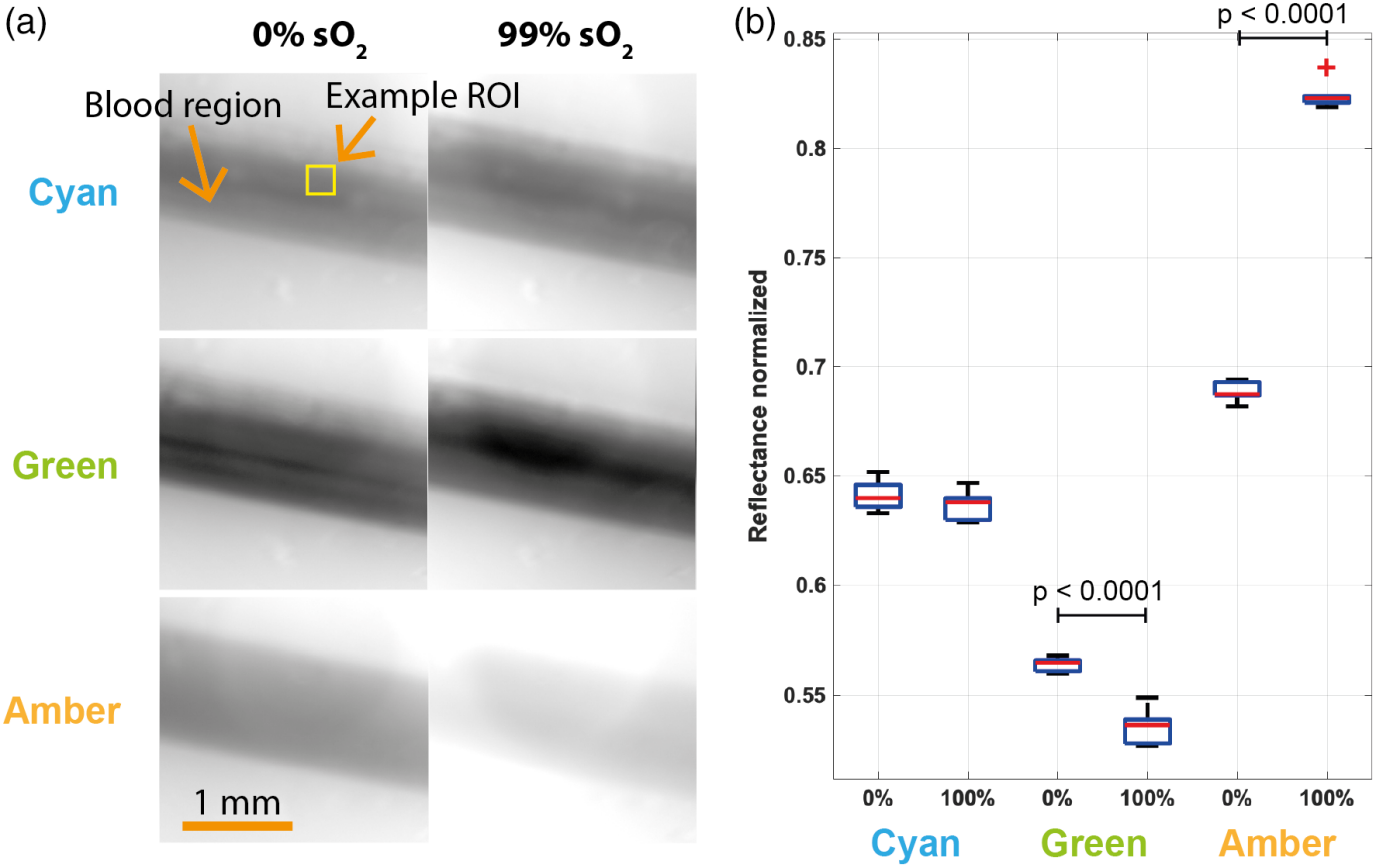
Multispectral LED-based imaging in blood phantoms. (a) The normalized images at 0% and 99% ${\mathrm{sO}}_{2}$ for cyan (top), green (middle), and amber (bottom) illuminations. Six ROIs in the blood region were analyzed. (b) Boxplot of the six ROI measurements illustrating the median and interquartile range of the normalized reflectance for the two values of blood oxygenation.

With the uniformity of illumination well understood, we then progressed to testing the LED-based MSI system using a phantom containing blood with differing oxygenation values. Images of the phantom taken under amber illumination clearly show the tubing containing the blood at high contrast compared to the background of the phantom [Fig. 5(a)]. The cyan LED showed little response to changing oxygenation and was statistically insignificant (Table 3), as would be expected as this was designed to target an isosbestic point of hemoglobin. The green LED reflectance [Fig. 5(b)] decreased with increasing oxygenation as expected (p<0.0001). The change in the reflectance was fairly small, only a 5% change, and this was expected based on the modeling. The amber LED reflectance increased with increasing oxygenation (p<0.0001) and showed a much greater change in reflectance due to the change in oxygenation of nearly 20%.

**Table 3:** The mean reflectance of the phantom at 0% and 99% ${\mathrm{sO}}_{2}$ was calculated based on six ROI and the ratio of these reflectance’s, the t-value, and the p-value were calculated.

	Mean reflectance		Ratio	Statistical analysis	
Illumination	0% ${\mathrm{sO}}_{2}$	99% ${\mathrm{sO}}_{2}$	99% ${\mathrm{sO}}_{2}$/0% ${\mathrm{sO}}_{2}$	t-value	p-value
Cyan	0.641	0.637	0.994	1.045	0.321
Green	0.564	0.536	0.950	7.990	<0.0001
Amber	0.689	0.825	1.198	42.936	<0.0001

### 3.3 LED-Based MSI Capillaroscopy Shows Morphological, and Oxygenation Metrics can be Extracted

**Fig. 6:**
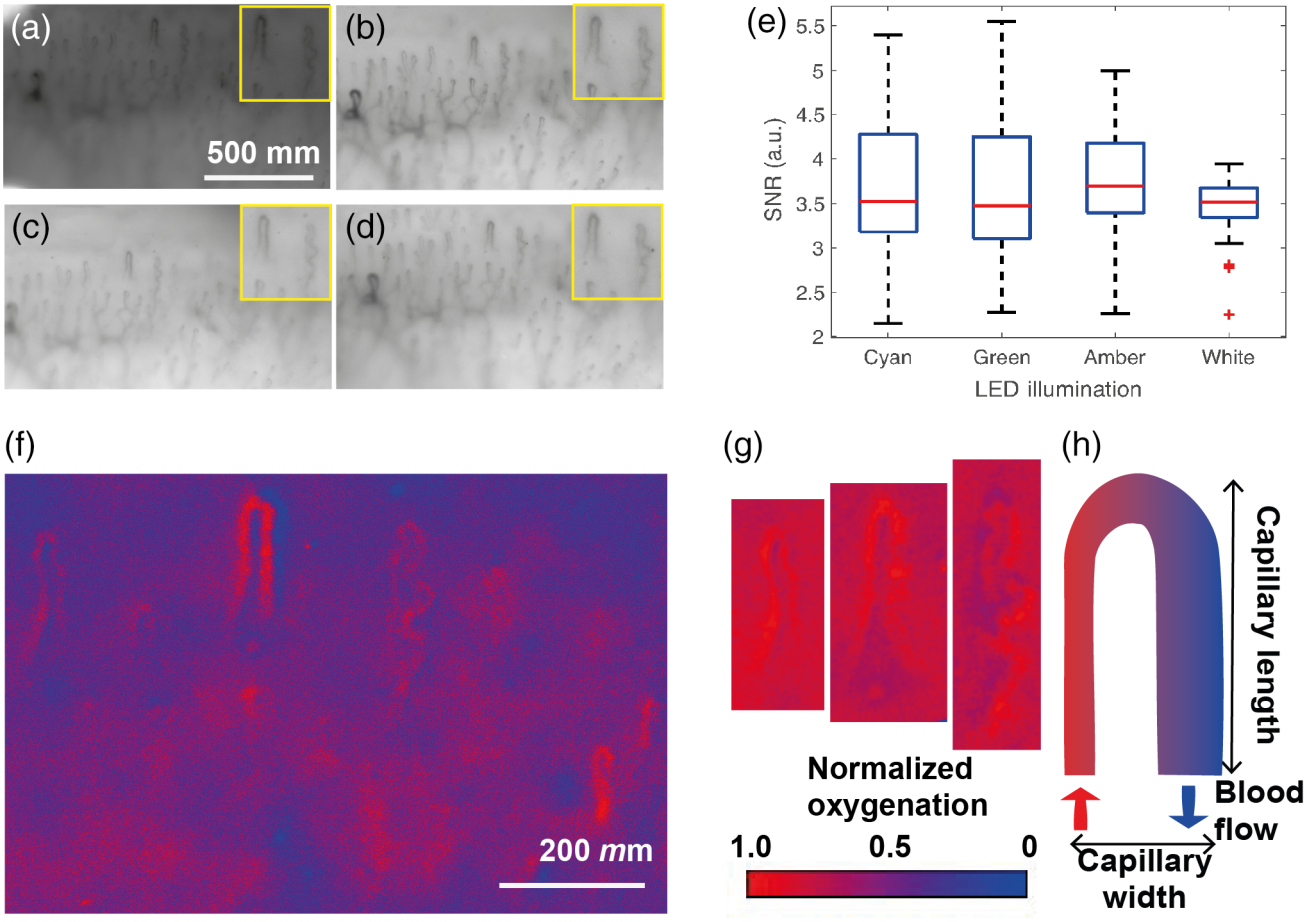
Multispectral LED-based nailfold capillaroscopy: (a)–(d) examples of capillary images taken using the different LEDs in the same finger. The yellow box in the right of each image shows an enlarged image of two capillaries. (e) Twenty-three capillaries were chosen from three nailfolds to analyze the image SNR based on illumination properties. The SNR of the capillaries imaged was similar for the four types of illumination used. (f) Select capillaries from an image of the fourth finger’s nailfold were aligned. (g) A relative oxygenation map was generated based on the ratio of the amber and cyan illuminated images, normalized to a maximum of 1.0. Noise and artifacts in the image are due to the alignment of the original images. (h) Two of the most common capillary dimensions measured are the capillary’s width and length. Also demonstrated is the hairpin shape that indicates healthy capillaries.

Finally, the multispectral LED ring light was used to illuminate the nailfolds of a healthy volunteer (MT-W). Each nailfold was coated in castor oil for refractive index matching and illuminated with each of the four different LEDs: cyan [Fig. 6(a)], green [Fig. 6(b)], amber (Fig. 6(c)], and white [Fig. 6(d)]. A subset of capillaries (n=23) from three different nailfolds (second, third, and fourth finger) of the first author was chosen for further analysis to determine the image SNR. The SNR of the capillaries did not vary significantly based on the LED illumination used [Fig. 6(e)].

For illustration purposes, a spatial map of the relative oxygenation of the nailfold was calculated based on the ratio of the amber to cyan illumination images after image alignment, either across the entire image [Fig. 6(f)] or alignment of selected individual capillaries [Fig. 6(g)]. In both methods, the relative oxygenation map was normalized to the maximum value. Although this approach gives only a relative oxygenation indication and a ground truth is not available from the *in vivo* nailfold scenario, the image does indicate the presence of oxygenated hemoglobin, as would be expected. The two-wavelength calculation also shows higher SNR than using all three wavelengths (Fig. S8 and Table S3 in the Supplementary Material). Absorption arising from other biomolecules in surrounding tissue leads to a nonzero background outside of the capillary structure due to the relatively simple ratiometric analysis applied here.

The loop morphology synonymous with nailfold capillaries is easily visualized in all illumination types, suggesting that narrowband illumination at select wavelengths is comparable with white light video capillaroscopy. Morphological properties of length and width [Fig. 6(h)] were extracted from all analyzed capillaries (Table 4). There was no significant difference in these dimensions based on the illumination LED at the 95% confidence level, and all measured values were consistent with the literature.^43^

**Table 4:** Comparison of morphological features of the capillaries between LEDs and to the literature. Comparisons are made using 23 capillaries measured on 3 fingers. The mean and standard error (SE) are given for each illumination type. These are compared with a reported range of literature values.

Illumination	Length (mean ± SE μm)	Width (mean ± SE μm)
Cyan	145.8 ± 7.9	35.5 ± 9.4
Green	170.8 ± 7.2	39.8 ± 2.4
Amber	164.0 ± 8.0	34.7 ± 1.8
White	157.5 ± 6.9	37.0 ± 2.2
Literature values^43^	146–270	32–46

## 4 Discussion

MSI has the potential to improve our understanding of how blood oxygen saturation relates to microvascular deformation in SSc and could improve disease diagnosis, monitoring, and treatment.^9^^,^^10^ Here, an LED-based MSI approach was designed, characterized and integrated into a nailfold capillaroscopy system for proof of principle testing. The system maintained the existing white light imaging capability and was able to provide the standard morphological assessments, while showing sensitivity to changing oxygenation status. The results make an important first step towards developing oxygen enhanced capillaroscopy techniques in the future. Simultaneous information on these metrics could provide clinicians with valuable insight into the functionality of the peripheral microvasculature in diseases such as SSc.^8^^,^^44^

The optical output of the LED ring light was modeled to determine spatial illumination uniformity and these results were compared with experimental measurements. There was some asymmetry, likely due to the imperfect experimental alignment of the LEDs, which reduced the overall uniformity of the illumination. To improve uniformity in future designs, 3D-printed receptacles could be created to receive the LEDs into known positions based on the modeling outputs. Furthermore, a diffuser could be integrated beneath the LED ring, although this would reduce the overall illumination power. The cyan LED showed particular disparities between measured and modeled results. Varying the angle of incidence of the LEDs and viewing angle of the LEDs in simulations showed that differences in alignments can affect the intensity drop-off of the ring lights. The drop-off of the model using a 22.3-deg incidence angle instead of 32.3-deg (a difference of 10 deg) better approximated the measured power drop-off of the LEDs in the z axis. Further work could experimentally measure and validate the angular intensity of the LEDs. The LEDs also exhibited variation in illumination power as a function of time after switching on, as expected. This added a source of variation in illumination power, which had to be controlled. Such effects could be mitigated in several ways, including timed strobing, where temperature variations might be controlled by timing the illumination, or selecting higher performance LEDs with improved temporal stability.

The changes in reflectance for the three-narrowband LEDs for different values of ${\mathrm{sO}}_{2}$ were consistent with the wavelength-dependent absorption of hemoglobin that was modeled based on spectral reflectance. Nonetheless, there remain some limitations to our study that should be addressed in future work. First, the potential for absolute oxygenation quantification in the range expected to be of relevance for SSc% (80 to 100%) should be established. Use of a tissue-mimicking phantom more similar to the morphology of nailfold with tunable oxygenation and flow rate properties would allow a more accurate assessment of the system, as greater reflectance changes would be expected to occur due to the presence of fewer blood cells.^45^^,^^46^ The absence of scatterers in the phantom and the channel depth of around 30  μm enables better contrast for the current proof of concept but is not representative of human tissue. Future developments in the phantom could better mimic the size of the blood vessels (both in depth and width) as well as the absorption and scattering of surrounding tissue. It would also be valuable to construct a tissue mimicking phantom that accounts for variations in skin pigmentation through integration of melanin. Accurate quantification is particularly important in the disease setting to discern the range of oxygenation values considered abnormal. Second, capillaries were visualized but at relatively low contrast, which could be improved by higher dynamic range imaging. By taking multiple images with varied exposure times, higher contrast in the capillary or blood region could be achieved, which may help to differentiate the anticipated small changes in oxygenation that would be seen in a realistic clinical setting. The potential downside of using high dynamic range techniques is that they often require longer exposure times (sometimes up to 10 times) resulting in motion artifacts and lower frame rates if used in video capillaroscopy. Finally, in the present study, we had to allow for stabilization of the LEDs used for illumination so the different colors were measured individually at high frame rate, but were not interleaved to enable real-time MSI as would be desired in a clinical application. Future work should examine the stability of a wider range of LEDs at the desired center wavelengths to select more amber and green LEDs akin in stability to the cyan LED used.

In summary, a system has been developed towards imaging oximetry in the nailfold, showing initial promise. The ring light was made from commercially available LEDs and a PCB. For a future clinical deployment, the whole system is envisaged to be mounted on a [x,y,z] translation stage for repeatable scanning of the nailfold and strobing the LEDs to allow spectral imaging. Further developments to improve illumination uniformity, temporal stability, and dynamic range could lead to a system suitable for application in management for patients with SSc.

## Code, Data, and Materials Availability

All data and arising code for analysis will be made available at: https://doi.org/10.17863/CAM.84746.
