# Supplementary material for: Multispectral imaging of nailfold capillaries using light-emitting diode illumination
Source: J Biomed Opt. 2022 Dec 12;27(12):126002. doi: 10.1117/1.JBO.27.12.126002 (PMC9743620; doi:10.1117/1.JBO.27.12.126002)
Supplement: Supplementary file 1 [file JBO_027_126002_SD001.pdf]

Supplementary Material for:

Multispectral imaging of nailfold capillaries using LED illumination

Michaela Taylor-Williams,[1,2] Stephen Mead,[1,2] Travis W Sawyer,[3] Lina Hacker,[1,2] Calum Williams,[1,2] Michael Berks,[4] Andrea Murray,[5] Sarah E Bohndiek[1,2] *

[1] Department of Physics, Cavendish Laboratory, University of Cambridge, JJ Thomson Avenue, Cambridge, CB3 0HE, UK

[2] Cancer Research UK Cambridge Institute, University of Cambridge, Robinson Way, Cambridge, CB2 0RE, UK

[3] Wyant College of Optical Sciences, University of Arizona, 1630 E University Blvd, Tucson, 85719, USA

[4] Quantitative Biomedical Imaging Laboratory, Division of Cancer Sciences, University of Manchester, Manchester M13 9PL, UK

[5] NIHR Manchester Biomedical Research Centre, University of Manchester, Manchester M13 9PL, UK.

Electrical Design and Fabrication

Each of the four types of LED required a constant current source to maintain constant radiant power when the LED was switched on. A circuit was assembled in-house to drive this constant current when triggered by a control signal (Fig. 1). The digital on/off control to switch the LEDs was an adjustable voltage signal (on = 3.3 V, off = 0 V). The circuit converted the control voltage source to a current sink. The green LEDs were connected serially in a string of eight between this sink and an auxiliary 20 V power supply. Their drive current was maintained at 50 mA (nominal), fixed by the values of resistors R2, R3, and R4, and the base-emitter voltage (0.65 V nominal) at NPN transistor Q1. Similarly, strings of amber, cyan, and white LEDs were driven at 50 mA, 20 mA, and 20mA, respectively.

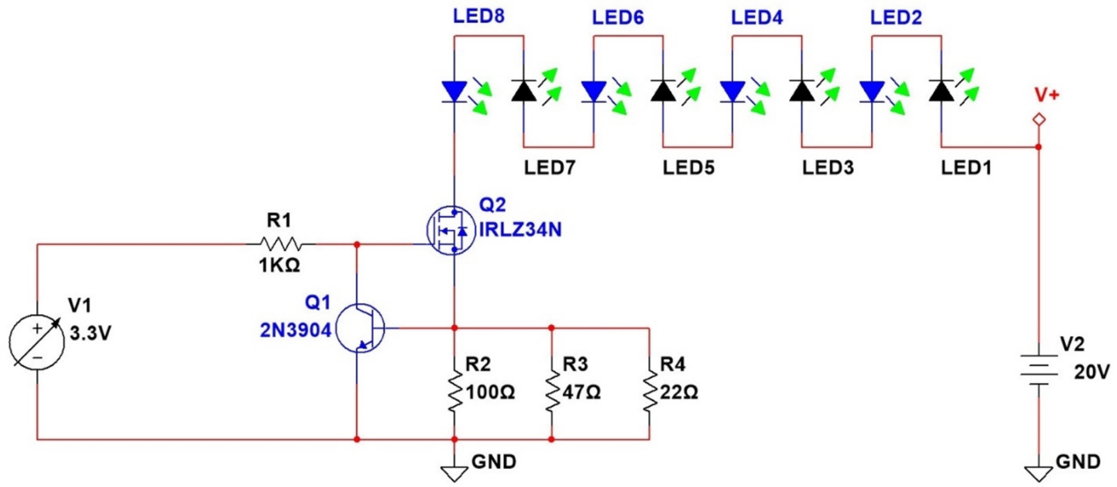

Supplementary Fig. 1: Constant current driver for LED string. Voltage source V1 represents the control voltage, shown here in its "on" state of 3.3 V. The LED output power could be lowered by reducing the level of V1.

Measurement of LED optical power over time

The LED ring array was fixed co-axially above a photodiode (Thorlabs S120C), so that the underside of the p.c.b. was positioned a distance Z1 = 67.2 mm above the photodiode's sensor surface (Fig. 2f). The output of the photodiode was monitored by an optical power meter (Thorlabs PM400). The array of green LEDs was connected according to Supplementary Fig. 1. The control

voltage was applied, and simultaneously, logging of the irradiance at the photodiode sensor commenced. The irradiance was recorded at a sample rate of 1 sample per second for a period of 30 minutes. The experiment was repeated for the cyan and amber LEDs separately and a plot was drawn to compare the variation of irradiance over time (Supplementary Fig. 2).

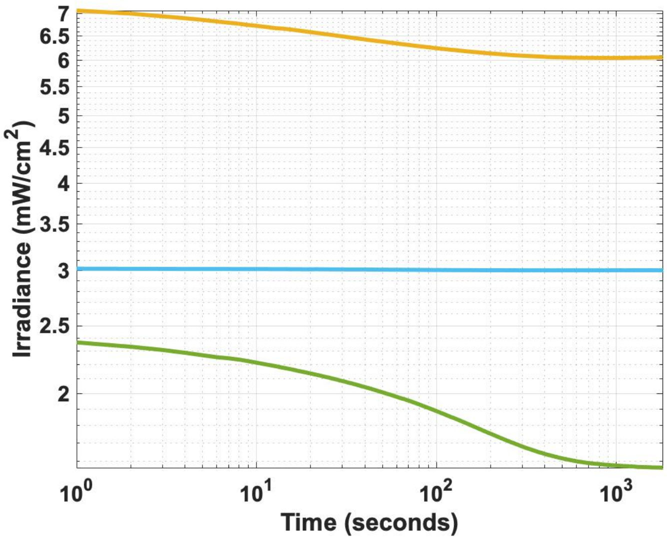

Supplementary Fig. 2: Measurement of LED irradiance over time by color from 0 to 1800 seconds. Each LED color is indicated by the color of the line in the graph. Both the time and irradiance are plotted on a logarithmic scale.

Spectral Characterization

The spectral profile of the LEDs and the linearity of the sensors used in the experimental systems were confirmed using the experimental system illustrated in Supplementary Fig. 3. The relative irradiance of each LED type was measured using a spectrometer (Avantes AvaSpec-ULS2048-USB2-FCPC), which was USB-connected to a computer running the manufacturer's software (AvaSoft v8). The spectrometer was first calibrated against the reference spectral profile of a halogen light source (Avantes AvaLight-HAL-CAL-MINI), which was connected to the spectrometer using a multimode fiber patch cable with 600 μm core (Thorlabs M134-L02). Following calibration, the patch cable was used to connect the spectrometer to the output port of

an integrating sphere (Thorlabs IS200). Each LED was successively fitted to the input port of the sphere and driven at a nominal constant current recommended in its datasheet. A silicon photodiode (Thorlabs SM05PD1B) at a second output port was used to determine when the LED peak output had stabilized following warm-up. The photodiode output was converted to a measurable voltage using a transimpedance amplifier (Thorlabs AMP100). The spectral profile of the LED was stored as a .csv data file and plotted using MATLAB software (Supplementary Fig. 4).

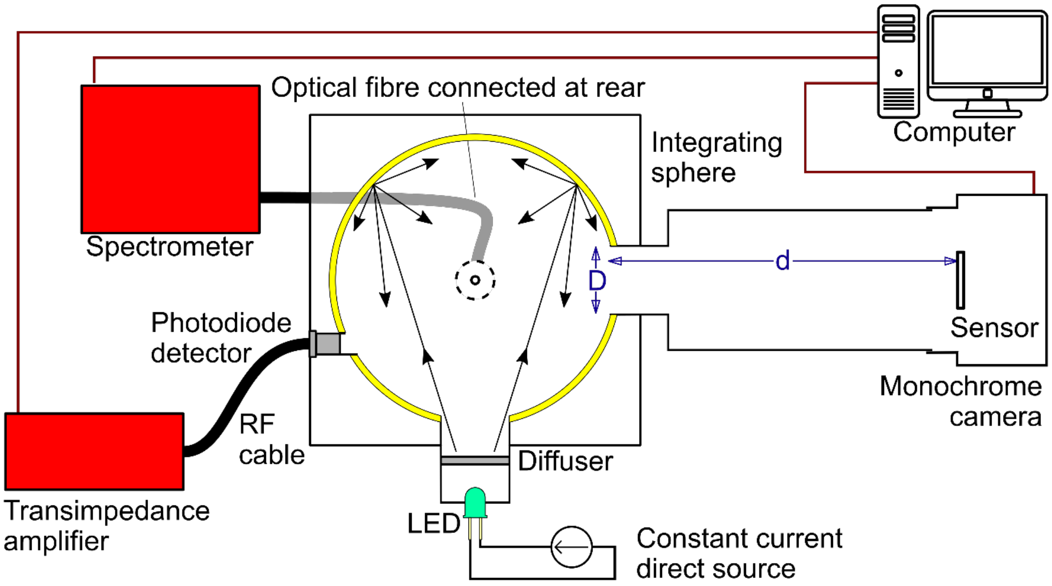

Supplementary Fig. 3: Experimental setup to measure the spectral profile of the LEDs and sensitivity of the monochrome camera. D=10.9 mm, d=108 mm.

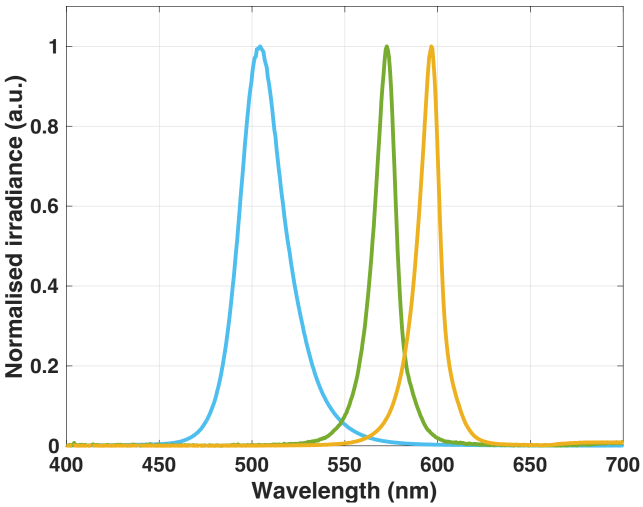

Supplementary Fig. 4: Measured spectral profiles of the LEDs. Each LED color is indicated by the color of the line in the graph.

The spectral properties of the cyan, green, and amber LEDs are summarized in Supplementary Table 1. The measured spectral properties were similar to those of the datasheet for the LEDs. The only one that showed a slight difference was the amber since the peak is slightly off (6 nm) but still within the tolerance given on the datasheet.

Supplementary Table 1: The measured spectral properties of the LEDs are summarized for comparison to the datasheet values in Table 1.

**LED**	**Spectral Peak Measured (nm)**	**FWHM (nm)**
Cyan	504.4	27.7
Green	572.6	13.5
Amber	596.6	12.9

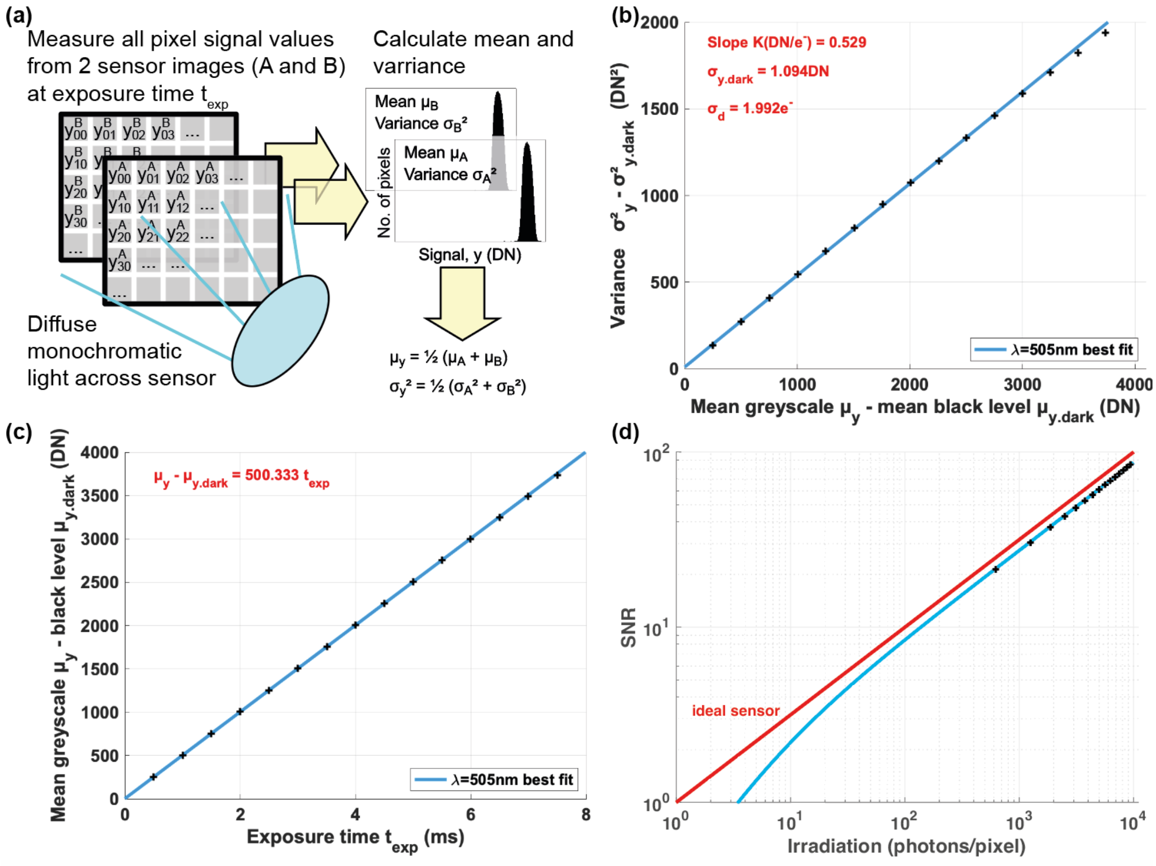

Supplementary Fig. 5: Sensor characterization (Basler a2A3840-45umPRO) using a cyan LED (peak wavelength λ=505 nm) source. (a) The sensor was illuminated uniformly by diffuse LED light, and the signal mean and variance was calculated for two images at each exposure time; (b) photo-induced variance was then plotted against the mean photo-induced greyscale values, (c) mean sensor greyscale value was plotted against image exposure time, and (d) signal-to-noise ratio of the sensor was plotted to determine its noise equivalent irradiance (SNR=1).

The output port of the sphere acted as a disk-shaped homogeneous illumination source of diameter 10.9 mm. The camera sensor (without lens) was positioned coaxially with this port at a distance of 108 mm from the sphere's opening. Basler Pylon Viewer v6.2 was used to set the camera parameters and capture the raw images. A signal offset of 24 DN was added to the camera signal span of 0 to 4095 DN to stop the noise level at zero irradiance from clipping the lower boundary of the measurement range.

It was assumed that, for a fixed irradiance, the sensor's mean digital output signal value, μ_y, was linearly proportional to the number of incident photons per pixel, μ_p:[33]

$$\mu_y - \mu_{y.dark} = K\, \eta\, \mu_p\,, \qquad (1)$$

where $\mu_{y.dark}$ is the mean signal value with no light incident on the sensor, K is the system gain, and η is total quantum efficiency. It was also assumed that the variance of the output noise, σ_y^2, was proportional to the mean output signal multiplied by the gain:[33]

$$\sigma_y^2 - \sigma_{y.dark}^2 = K(\mu_y - \mu_{y.dark}), \qquad (2)$$

where $\sigma_{y.dark}^2$ is the variance of the output noise with no light on the sensor. The histogram of the pixels' greyscale values was examined in real-time, and the camera exposure time increased until ≥99% of the pixels were below saturation. Two sequential greyscale images were captured at this exposure time. The LED light source was then turned off, and two dark reference images were captured at the same exposure time. The exposure time was decreased in steps of 0.5 ms and the two greyscale and two dark image capture process repeated at each step. The experiment was conducted at constant room temperature. The system gain of the camera was determined to be 0.529 from the slope of this photon transfer curve (Supplementary Fig. 5).

The linearity of the Grasshopper sensor (FLIR Grasshopper Camera GS3-U3-41C6M-C) was checked using the same set-up as Supplementary Fig. 3, but a white LED (Dolan-Jenner MI-LED-UK-A7) was coupled into the integrating sphere using a fiber optic, instead of the individual LEDs. The intensity of the light from the LED was varied at 13 different intensities, validated with the reference spectrometer. The area under the curve of the spectrometer data was taken at each illumination point and results were normalized to the highest illumination intensity. The sensor response was evaluated using the SpinView v2.2.0.48 software package (Teledyne FLIR) to find the mean greyscale value. The process was repeated ten times to find the average sensor response and illumination intensity (Supplementary Fig. 6), which was demonstrated to be linear with an R2= 0.9949.

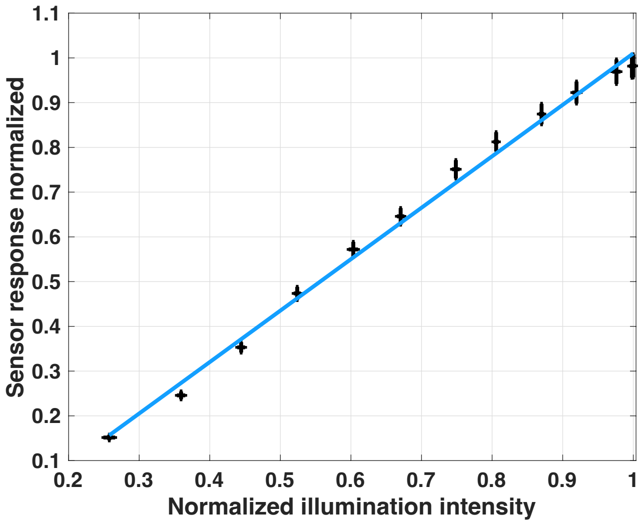

Supplementary Fig. 6: Sensor characterization (FLIR Grasshopper) using a white LED source. (a) The sensor was illuminated uniformly by LED light, and the signal mean and variance were calculated for two images at each illumination intensity.

LED Modeling of Cyan with different viewing angles and angles of incidence

The cyan LED system model was adjusted to better understand the difference in illumination profiles of the system. This was done two ways: the viewing angle was decreased to half of the initial model (from 15° to 7.5°); and the angle of incidence, or angle made between the LEDs and the z axis, was decreased by 10° (from 32.3° to 22.3°). The resulting profiles are shown in Supplementary Fig. 7 and the RMSE in the Z and X planes are summarized in Supplementary Table 2. The decrease in viewing angle (Remodel A) resulted in the maximum intensity peaking at 7.25 mm in front of the object plane compared to 5.875 mm when measured, or 18.38 mm in the initial model. The model with a decreased angle of incidence (Remodel B) peaked at 14.38 mm, but the overall shape of the power drop-off was more similar to the measured results, as indicated in the RMSE values calculated along the z axis in Table 2. The RMSE of the initial model was 0.235, while the remodeled systems A and B were 0.340 and 0.137, respectively, indicating that A was slightly worse than the original model, while B was an improvement. The

continued discrepancy could be due to misalignment of the measuring systems or asymmetries of LED placement in the experimental system. The same trend could be seen in the RMSE of the x axis, which indicates the decreased angle of incidence better models the built system than the original and remodel, with a smaller viewing angle.

Supplementary Table 2: The Cyan system was remodeled two different ways including varying the viewing angle and the angle of incidence of the LEDs and the associated RMSE values, relative to the measured system, calculated in both the z and x axis.

Cyan Systems Compared	Viewing Angle	Angle of incidence	Z axis RMSE	X axis RMSE
Initial Model	15°	32.3°	0.235	0.042
Remodel A	7.5°	32.3°	0.340	0.310
Remodel B	15°	22.3°	0.137	0.036

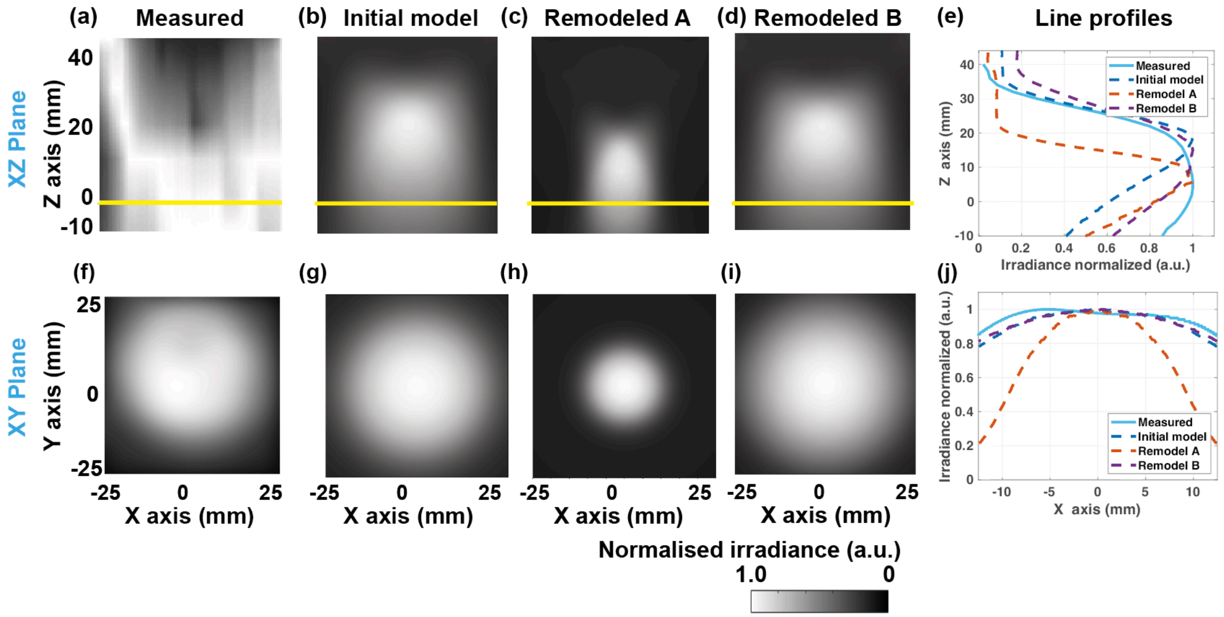

Supplementary Fig. 7: The (a) measured cyan system's drop off along the z axis was compared to the (b) initial model and two separate remodels that varied the (c) viewing angle and (d) angle of incidence. (e) The associated profiles are shown to illustrate this comparison. The same was done in the x axis (f-j).

Image reconstruction of the nailfold capillaries

Two and three wavelength reconstruction of the relative oxygenation in the nailfold capillaries was explored by calculating three different images using the narrowband images collected, after image processing performed as described in Section 2.7. Three different images were calculated by the following equations:

$$\frac{I_{amber}}{I_{cyan}} \quad (3)$$

$$\frac{I_{green}}{I_{cyan}} \quad (4)$$

$$\frac{I_{amber}-I_{green}}{I_{cyan}} \quad (5)$$

The resulting images are shown in Supplementary Fig. 8. For each of these images the SNR, signal, background signal, and background noise were calculated (Table 3). The image calculated using Eqn. 3 had the highest SNR compared to the other two methods, in part due to the lower noise.

Supplementary Table 3: The average SNR of the different image analysis methods are shown below. The average signal, background signal, and noise (calculated as a standard deviation) are also shown.

Image calculation method	Average SNR	Average Signal	Average Background Signal	Average Noise (Standard deviation)
Amber/Cyan	0.794	2.147	1.354	0.157
Green/Cyan	0.655	2.663	2.008	0.247
(Amber-Green)/Cyan	0.683	4.246	1.354	0.367

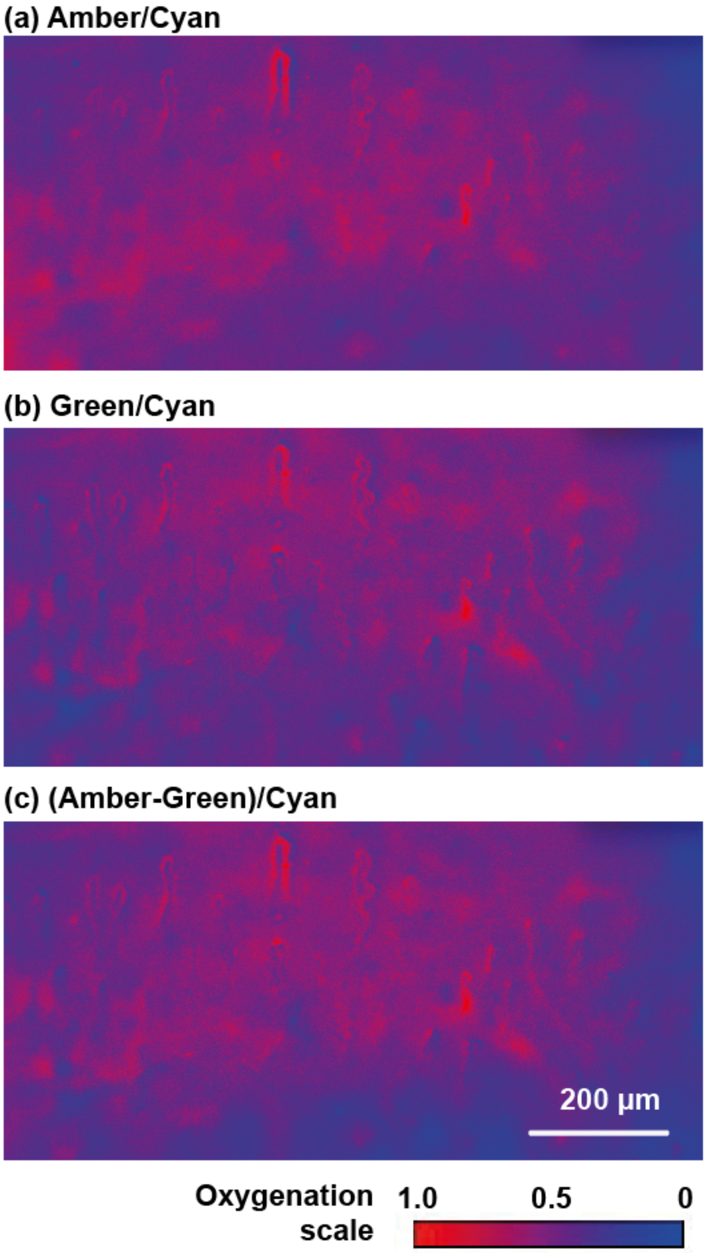

Supplementary Fig. 8: Illustration of using different methods to calculate the relative oxygenation of the nailfold: (a) amber divided by cyan as given by eqn. 3, (b) green divided by cyan as given by eqn. 4 and (c) the difference between amber and green images that are then divided by the cyan image as given by eqn. 5.
